# Tetrameric Cyclic Double Helicates as a Scaffold for a Molecular Solomon Link**

**DOI:** 10.1002/anie.201302634

**Published:** 2013-05-06

**Authors:** Jonathon E Beves, Christopher J Campbell, David A Leigh, Robin G Pritchard

**Affiliations:** School of Chemistry, University of Edinburgh, The King's BuildingsWest Mains Road, Edinburgh EH9 3JJ (UK); School of Chemistry, University of ManchesterOxford Road, Manchester M13 9PL (UK)

**Keywords:** catenanes, chemical topology, cyclic helicates, molecular knot, self-assembly

**Figure 1:**
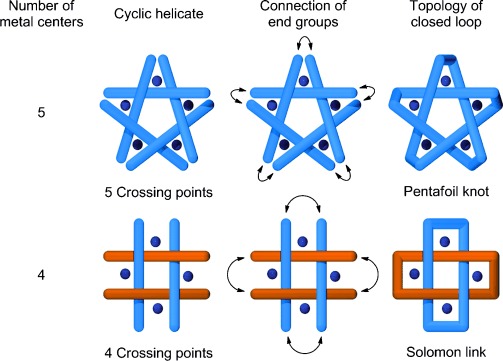
Ring-closing cyclic metal double helicates for the formation of topologically complex molecules. A pentameric circular double helicate is the scaffold (five crossings) required for a pentafoil knot,[9] and a tetrameric circular double helicate (four crossings) the basis for a Solomon link.[2b,d]

A Solomon link, colloquially termed a “Solomon knot” (a 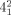 link in Alexander–Briggs notation[1]), is a topology of two interwoven rings that cross each other four times in the simplest representation (Figure 1).[2] Such doubly-entwined [2]catenanes are still rare,[3–5] with only two small-molecule examples with wholly organic backbones reported[4,5] to date. The Solomon link is the most complex topology to have been produced[4] using Sauvage’s pioneering route[6] of generating higher order interlocked structures through the connection of the termini of linear double-stranded metal helicates. In principle,[2b,d] cyclic double helicates[7] can provide the crossings required for a range of topologies, while simultaneously positioning connecting sites in close proximity to aid the macrocyclization reactions that can be problematic when employing long linear helicates[8] (Figure 1). A small-molecule pentafoil knot (five crossings) was recently prepared using a pentameric circular helicate scaffold.[9] Here we report on the use of a tetrameric circular helicate as the basis for a Solomon link, illustrating the general utility of this approach for the assembly of complex molecular topologies.

**Scheme 1:**
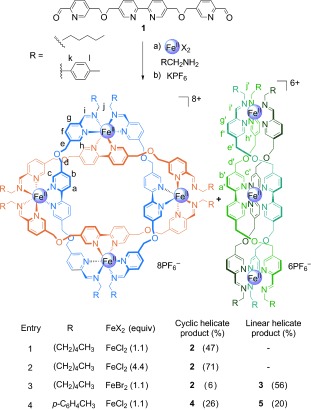
Synthesis of cyclic and linear iron(II) helicates. Reaction conditions: a) FeX_2_, RCH_2_NH_2_, DMSO, 60 °C, 24 h; b) excess KPF_6_ (aq). DMSO=dimethyl sulfoxide.

The ligand used in our earlier synthesis of a pentafoil knot[9] was based on a tris(bipyridine) motif employed[7a,b,d] by Lehn to assemble penta- and hexameric cyclic helicates, but with both outer bipyridine units replaced by 2-formylpyridine groups that could condense with amines to form imines and generate tris(bidentate) ligand strands. As well as providing a convenient way of connecting metal binding components, imine bond formation is reversible, imparting an ‘error checking’ mechanism during the assembly process.[10] Incorporating an additional oxygen atom in the ethylene spacer between each bipyridine group of Lehn’s tris(bipyridine) ligand led to cyclic tetrameric helicates.[7b] Accordingly, in an attempt to generate the four crossings required for a Solomon link, we introduced a similar structural change to the ligand used in the pentafoil knot synthesis in the form of **1** (for the synthesis of **1** see the Supporting Information) and investigated its coordination chemistry with primary amines and Fe^II^ salts (Scheme 1).

**Figure 2:**
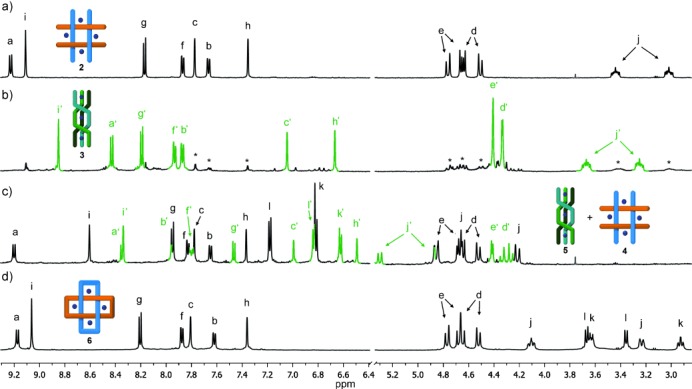
^1^H NMR spectra (CD_3_CN, 500 MHz) for a) cyclic tetramer 2, b) linear triple helicate 3 (green, signals marked * correspond to trace amounts of 2), c) a 1:1 mixture of cyclic tetramer 4 (black) and linear triple helicate 5 (green), and d) Solomon link 6. All spectra were recorded in the presence of AgPF_6_ (0.1 equiv) to remove traces of chloride ions and sharpen the signal of H_a_. For spectra collected in the absence of AgPF_6_, see the Supporting Information, Figures S10–S12. Signal assignments refer to the lettering shown in Schemes 1 and 2.

The reaction of **1** with *n*-hexylamine and FeCl_2_ (DMSO, 60 °C, 24 h, Scheme 1)[8] produced an intensely colored purple solution typical of low-spin iron(II) tris(diimine) complexes. After 24 hours, the product was isolated in 47 % yield as the hexafluorophosphate salt **2** by precipitation with aqueous KPF_6_. Electrospray ionization mass spectrometry (ESI-MS; see the Supporting Information, Figure S1) revealed that **2** was a metal–ligand tetramer with the formula [Fe_4_L_4_](PF_6_)_8_][11] (L=bis(imine) ligand resulting from the condensation of **1** with two molecules of *n*-hexylamine). ^1^H NMR spectroscopy (Figure 2 a) indicated that **2** was highly symmetrical, with the splitting of the diastereotopic CH_2_-O-CH_2_ protons consistent with the chiral (racemic) helicate topology shown in Scheme 1. The yield of **2** was increased to 71 % (yield of isolated product) when employing 4.4 equivalents of the iron(II) salt (see the Supporting Information, Figure S9).

The formation of the tetrameric cyclic helicate was not limited to the use of FeCl_2_ as the iron(II) salt (Scheme 1), both Fe(BF_4_)_2_ and Fe(ClO_4_)_2_ also produced **2**, although in significantly lower yields (see the Supporting Information, Figure S13) and contaminated with other polymeric and oligomeric by-products. When FeBr_2_ was employed as the iron source, a different main product was obtained (Scheme 1), which was identified as the linear trinuclear triple helicate ([Fe_3_L_3_]^6+^) **3** by ^1^H NMR spectroscopy (Figure 2 b) and ESI-MS (see the Supporting Information, Figure S14). A linear triple helicate with a lifetime of a few minutes was previously observed as an intermediate during the formation of pentameric cyclic helicates using Lehn’s tris(bipyridine) ligand.[7d] While **3** is a much longer-lived species, it is not clear whether this is because the linear triple helicate is particularly stable as the bromide salt, or whether the assembly/disassembly/rearrangement of the various linear and circular helicates and oligomers is markedly slower using FeBr_2_, perhaps as a result of their limited solubility.

Substituting *n*-hexylamine for 4-methylbenzylamine in the reaction of **1** with FeCl_2_ gave a mixture of two species (Figure 2 c), identified by ESI-MS (Supporting Information, Figures S3 and S5) as the cyclic tetramer **4** and the linear triple helicate ([Fe_3_L_3_]^6+^) **5** (Scheme 1). Using our standard reaction protocol with an initial concentration of **1** of 2.2 mm, the ratio of **4**/**5** was approximately 1:1, however the distribution of cyclic-double-helicate/linear-triple-helicate was significantly altered by small variations in concentration: using an initial concentration of 8.8 mm of **1**, more than 95 % of the reaction product was the higher order (four ligands, four metal ions) circular helicate **4** after 24 hours, whereas starting with a concentration of 0.55 mm of **1**, the reaction produced more than 85 % of the lower nuclearity (three ligands, three metal ions) linear helicate **5** over the same time period (Supporting Information, Figure S15).[12] In contrast, the yield of the analogous *n*-hexylamine-derived cyclic tetramer **2** was essentially invariant over this concentration range and no linear triple helicate was observed, illustrating the influence that subtle changes in the ligands can have over the outcomes of the self-assembly reactions.

**Scheme 2:**
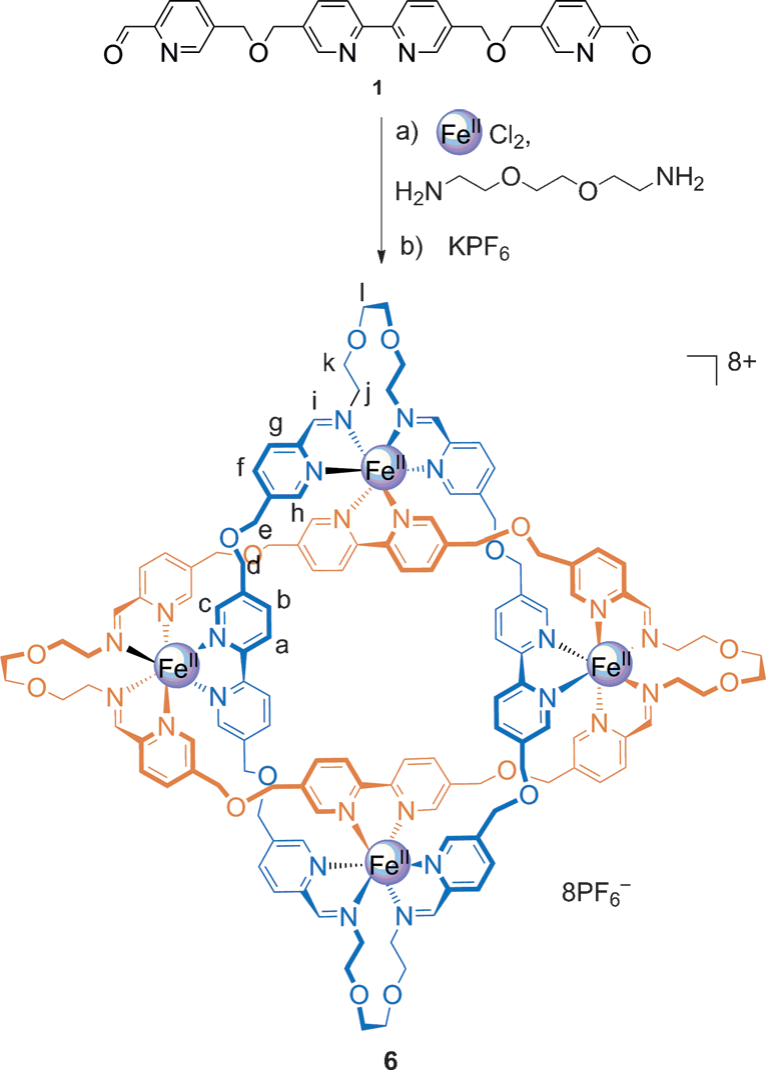
Synthesis of molecular Solomon link 6. Reaction conditions: a) FeCl_2_, 2,2′-(ethylenedioxy)bis(ethylamine), DMSO, 60 °C, 24 h; b) excess KPF_6_ (aq), 75 % (over two steps).

In order to link the end groups of the open cyclic helicate to generate a Solomon link, we employed 2,2′-(ethylenedioxy)bis(ethylamine), a diamine that is stereoelectronically predisposed to adopt low-energy turns.[9] The reaction of **1** with the diamine and FeCl_2_ in DMSO for 24 hours, with subsequent anion exchange with aqueous KPF_6_, generated the Solomon link **6** in 75 % yield of isolated product (Scheme 2).[13]

**Figure 3:**
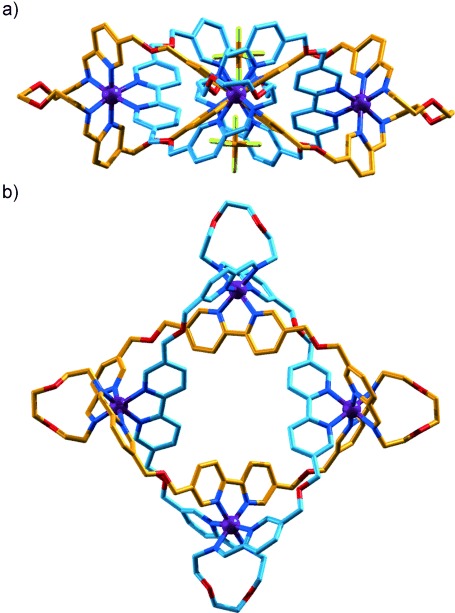
X-Ray crystal structure of Solomon link 6. a) Viewed in the plane of Fe^II^ ions (all but two PF_6_^−^ anions omitted); b) viewed from above the center of the macrocycle cavities (all PF_6_^−^ anions omitted). The C atoms of one ring are colored orange and of the other ring light blue, N: dark blue, O: red, P: brown, F: green, Fe: purple. Solvent molecules, H atoms, and PF_6_^−^ anions (other than the two shown in view (a)) are omitted for clarity. Fe–Fe distances [Å]: 10.590(6), 10.677(7), 10.590(6), 10.709(7), and Fe-Fe-Fe angles [°]: 90.05(4), 89.85(4), 90.20(4), 89.66(4). O-C-C-O torsion angles [°]: 70(3), 59(3), 67(3), 73(3). CH_a_⋅⋅⋅F distances [Å]: 2.72, 2.82, 2.74, 2.65, 2.78, 2.89, 2.99, 2.74, 2.96, 2.70, 2.86, 2.88, 2.85, 3.15, 2.95, 2.87.

The ^1^H NMR spectrum (CD_3_CN, 500 MHz, Figure 2 d) of **6** is very similar to that of the tetrameric cyclic helicate **2** derived from *n*-hexylamine (Figure 2 a), including the splitting pattern for the diastereotopic CH_2_-O-CH_2_ protons. ESI-MS (Supporting Information, Figure S7) confirmed that **6** had a structural formula consistent with a Solomon link. Single crystals of **6** suitable for X-ray crystallography were grown by slow diffusion of diethyl ether into a nitromethane solution of **6**, and the structure was confirmed by X-ray crystallography (Figure 3). The solid-state structure shows the two organic macrocycles interlocked by the four crossings that define the topology of a Solomon link. The iron atoms are close-to-coplanar and lie on the vertices of a square with Fe–Fe distances of just over 1 nm. Despite the high yield, as for the related pentafoil knot,[9] the octahedral coordination geometry of the iron(II) centers is amongst the most distorted [Fe(N-ligand)_6_] structures in the Cambridge Structural Database[14] (see the Supporting Information for details). The -OCH_2_CH_2_O- units in the linking group adopt close-to-gauche conformations (59–73°). Two PF_6_^−^ counter ions are positioned directly above and below the center of the helicate (Figure 3 a) and form bifurcated CH⋅⋅⋅F interactions with the eight H_a_ protons, which are particularly electron-poor because of the ligand coordination to the iron(II) dications (Supporting Information, Figures S16 and S17).

The one-pot synthesis of molecular Solomon link **6** assembles four iron(II) cations, four bis(aldehyde) and four bis(amine) building blocks to generate two interwoven 68-membered-ring macrocycles with four crossings in 75 % isolated yield. The assembly process for the tetrameric cyclic double helicate forms the basis for the Solomon link synthesis and is sensitive to structural changes in the amine, the concentration and the anion used (even though the reaction product is not the result of an anion-template mechanism). The synthesis of Solomon link **6** and the earlier pentafoil knot[9] show that cyclic helicates of different sizes can act as highly efficient and effective scaffolds for intricate molecular topologies.
