# Supplementary material for: Tetrameric Cyclic Double Helicates as a Scaffold for a Molecular Solomon Link
Source: Angew Chem Int Ed Engl. 2013 May 6;52(25):6464–7. doi: 10.1002/anie.201302634 (PMC4139000; doi:10.1002/anie.201302634)
Supplement: Supplementary file 1 [file anie0052-6464-sd1.pdf]

Angewandte Chemie
Eine Zeitschrift der Gesellschaft Deutscher Chemiker

Supporting Information
© Wiley-VCH 2013
69451 Weinheim, Germany

Tetrameric Cyclic Double Helicates as a Scaffold for a Molecular Solomon Link**

Jonathon E. Beves, Christopher J. Campbell, David A. Leigh, and Robin G. Pritchard*

anie_201302634_sm_miscellaneous_information.pdf

SI-Section 1: General Experimental Detail

Unless stated otherwise, all reagents and solvents were purchased from Aldrich Chemicals and used without further purification. Column chromatography was carried out using Silica 60A (particle size 35-70 µm, Fisher, UK) as the stationary phase, and TLC was performed on precoated silica gel plates (0.25 mm thick, 60 F254, Merck, Germany) and observed under UV light. NMR spectra were recorded on Bruker AV 400, and Bruker DMX 500 instruments. Chemical shifts are reported in parts per million (ppm) from low to high frequency and referenced to the residual solvent resonance. Coupling constants (*J*) are reported in hertz (Hz). Standard abbreviations indicating multiplicity were used as follows: s = singlet, d = doublet, t = triplet, dd = double doublet, q = quartet, m = multiplet, b = broad, ddd = doublet of double doublets. ^{1}H and ^{13}C NMR assignments were made using 2D-NMR methods (COSY, ROESY, TOCSY, HSQC, HMBC) and are unambiguous unless stated otherwise. Melting points (m.p.) were determined using a Sanyo Gallenkamp apparatus and are reported uncorrected. Low resolution ESI mass spectrometry was performed with a Finnigan LCQ-MS, Micromass Platform II or Waters Quattro Ultima LC-*MS*/*MS* mass spectrometers. High resolution ESI and FAB mass spectrometry were carried out by the mass spectrometry services at the University of Edinburgh and the EPSRC National Mass Spectrometry Service Centre, Swansea, UK.

SI-Section 2: Synthetic procedures

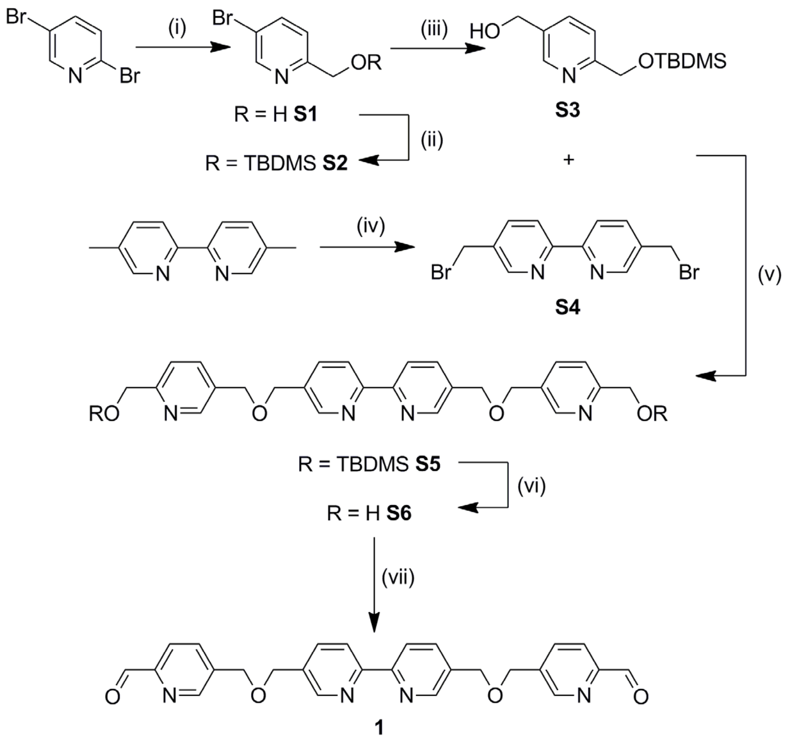

Scheme S1: Preparation of dialdehyde **1**. Reagents and conditions: (i) (a) *n*-BuLi, toluene, -78 ºC, 2 h then DMF, -78 ºC, 1 h; (b) $NaBH_4$, MeOH, RT, 1 h, 74 %; (ii) TBDMS-Cl, Et_3N, DMF, RT, 30 min, quant.; (iii) *n*-BuLi, Et_2O, -78 ºC, 1 h then DMF, -78 ºC, 1 h; (b) $NaBH_4$, MeOH, 0 ºC to RT, 15 min, 85 %; (iv) NBS, benzoyl peroxide, CCl_4, 80 ºC, 2 h, 41 %; (v) NaH, THF, 70 ºC, 18 h, 80 %; (vi) TBAF, THF, RT, 2 h, quant.; (vii) oxalyl chloride, DMSO, Et_3N, DCM, -78 ºC, 1.5 h, 53 %.

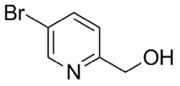

S1

S1 was prepared according to a modified literature procedure.[S1] To a stirred solution of 2,5-dibromopyridine (5.0 g, 21 mmol) in toluene (200 mL) at -78 ºC was slowly added *n*-butyl lithium (10 mL, 2.5 M in hexanes, 25 mmol). After stirring for 2 h at – 78 ºC, DMF (2 mL) was added and the reaction was stirred for an additional 1 h at – 78 ºC before being allowed to warm to r.t.. The mixture was quenched by addition of NH_4Cl (30 mL, sat. aq. solution). The aqueous layer was extracted with EtOAc (3 × 30 mL). The combined organic layers were dried ($MgSO_4$), filtered under gravity and the solvent removed in vacuo to give an orange solid. The crude solid was dissolved in MeOH (50 mL) and $NaBH_4$ (230 mg, 6.1 mmol) was added carefully. The solution was stirred for 1 h at r.t. before being quenched by addition of 2M HCl (50 mL), then basified by addition of K_2CO_3 (150 mL, 5 % aq. solution). The mixture was extracted

with DCM (3 × 50 mL) and the combined organic layers dried ($MgSO_4$), filtered under gravity and the solvent removed in vacuo to give a yellow oil that solidified upon standing (2.9 g, 74 %, over two steps). Spectroscopic data match those reported in the literature [S1]: ^{1}H NMR (400 MHz, $CDCl_3$) δ 8.62 (d, J = 2.1 Hz, 1H), 7.81 (dd, J = 8.3, 2.3 Hz, 1H), 7.20 (d, J = 8.6 Hz, 1H), 4.72 (s, J = 7.5 Hz, 2H), 3.42 (b s, 1H). LRESI-MS: m/z = 210.58 $[M+Na]^+$ (calcd. for $C_6H_6BrNNaO$, 209.95) The compound was of sufficient quality to be used in the next step without further purification.

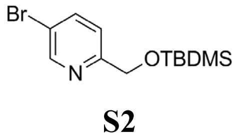

S2

Compound **S2** was prepared according to a modified literature procedure. [S2]
S1 (1.0 g, 5.4 mmol) and TBDMS-Cl (850 mg, 5.6 mmol) were suspended in DMF (4 mL) and Et_3N (1.1 mL, 8.0 mmol) was added. The reaction mixture was stirred for 30 min at RT before being quenched by addition of water (50 mL) and Et_2O (20 mL). The organic layer was washed with water (3 × 20 mL), then brine (20 mL), dried ($MgSO_4$), filtered under gravity and the solvent was removed in vacuo to give a yellow oil (1.6 g, quant.). Spectroscopic data match those reported in the literature [S2]: ^{1}H NMR (400 MHz, $CDCl_3$) δ 8.56 (d, J = 2.1 Hz, 1H), 7.82 (dd, J = 8.4, 2.3 Hz, 1H), 7.42 (d, J = 8.4 Hz, 1H), 4.77 (s, 2H), 0.95 (s, 9H), 0.10 (s, 6H). LRESI-MS: m/z = 302.42 $[M+H]^+$ (calcd. for $C_{12}H_{21}BrNOSi$, 302.06). The compound was of sufficient quality to be used in the next step without further purification.

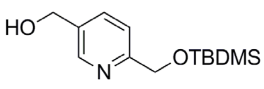

S3

S3 was prepared using a different synthetic route to that reported previously.[S3]
S2 (1.0 g, 3.3 mmol) was dissolved in Et_2O (30 mL) and cooled to -78 ºC. *n*-Butyl lithium (1.5 mL, 2.5 M in hexanes, 3.7 mmol) was slowly added to the reaction. The resulting mixture was stirred for 1 h at –78 ºC before DMF (0.8 mL) was added and the reaction was left to stir for a further 1 h at –78 ºC. Once warmed to r.t. the mixture was quenched with water (20 mL). The organic layer was washed with water (3 × 30 mL), then brine (30 mL), dried ($MgSO_4$), filtered under gravity and the solvent was removed in vacuo. The crude mixture was dissolved in MeOH (20 mL) and cooled to

0 ºC. $NaBH_4$ (0.11 g, 3.0 mmol) was slowly added. The solution was stirred for 15 min at r.t. before being quenched by addition of water (20 mL). The aqueous layer was extracted with EtOAc (3 × 10 mL). The combined organic layers were washed with water (3 × 30 mL), then brine (30 mL), dried ($MgSO_4$), filtered under gravity and the solvent was removed in vacuo The product was purified by flash chromatography (SiO_2, 4:1, DCM: EtOAc) to give **S3** as yellow crystals (710 mg, 85 %, over two steps). Spectroscopic data matched those reported in the literature [S3]: ^{1}H NMR (400 MHz, $CDCl_3$) δ 8.49 (s, 1H), 7.74 (dd, *J* = 8.1, 2.0 Hz, 1H), 7.52 (d, *J* = 8.1 Hz, 1H), 4.84 (s, 2H), 4.73 (d, *J* = 5.7 Hz, 2H), 0.96 (s, 9H), 0.12 (s, *J* = 3.0 Hz, 6H), LRESI-MS: *m/z* = 254.1 $[M+H]^+$ (calcd. for $C_{13}H_{24}NO_2Si$, 254.16).

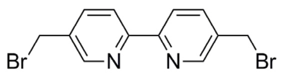

S4

S1 was prepared according to a literature procedure.[S4] A mixture of 5,5'-dimethyl-2,2'-dipyridyl (2.8 g, 15 mmol) and N-bromosuccinimide (5.4 g, 26 mmol) was heated for 30 min at 80 ºC in CCl_4 (150 mL). Benzoyl peroxide (30 mg, 0.12 mmol) was added and the reaction heated at 80 ºC for 2 h. The crude mixture was filtered, whilst, hot and the filtrate concentrated in vacuo. The crude product was recrystallized from THF to give **S4** as a colorless powder (2.5 g, 49 %). Spectroscopic data matched those reported in the literature [S4]: ^{1}H NMR (400 MHz, $CDCl_3$) δ 8.68 (d, *J* = 1.8 Hz, 2H), 8.40 (d, *J* = 8.2 Hz, 2H), 7.86 (dd, *J* = 8.2, 2.2 Hz, 2H), 4.54 (s, 4H). LRESI-MS: *m/z* = 341.33 $[M+H]^+$ (calcd. for $C_{12}H_{11}Br_2N_2$, 340.93).

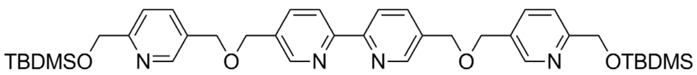

S5

S3 (170 mg, 0.61 mmol) was dissolved in THF (15 mL), NaH (27 mg, 60 % dispersion in mineral oil, 0.67 mmol) was added and the reaction stirred for 30 min at RT. **S4** (100 mg, 0.29 mmol) was added directly as a solid. The resulting orange mixture was heated at reflux for 18 h, cooled to r.t. and quenched by addition of MeOH (2mL), followed by addition of EtOAc (30 mL) and water (30 mL). The organic layer was washed with water (3 × 30 mL), and brine (30 mL), dried ($MgSO_4$), filtered under gravity and the solvent was removed in vacuo to give an off-white,

waxy solid (170 mg, 80 %). ^{1}H NMR (400 MHz, $CDCl_3$) δ 8.65 (d, *J* = 1.9 Hz, 2H), 8.50 (d, *J* = 1.9 Hz, 2H), 8.40 (d, *J* = 8.1 Hz, 2H), 7.83 (dd, *J* = 8.1, 2.0 Hz, 2H), 7.74 (dd, *J* = 8.0, 2.0 Hz, 2H), 7.53 (d, *J* = 8.1 Hz, 2H), 4.84 (s, 4H), 4.64 (s, 4H), 4.61 (s, 4H), 0.96 (s, 18H), 0.12 (s, *J* = 3.0 Hz, 12H) , ^{13}C NMR (126 MHz, $CDCl_3$) δ = 161.4, 155.8, 148.8, 148.4, 136.7, 136.6 x 2, 131.4, 121.1, 120.0, 70.1, 69.9, 66.2, 26.1, 18.5, -5.2. HRESI-MS: *m/z* = 687.3751 $[M+H]^+$ (calcd. for $C_{38}H_{55}N_4O_4$, 687.3762).

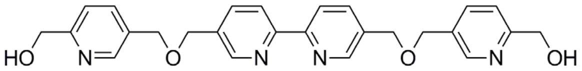

S6

S5 (160 mg, 3.0 mmol) was dissolved in THF (5 mL) and TBAF (1.85 mL, 1 M solution in THF, 1.85 mmol) was slowly added. The resulting solution was stirred for 2 h at r.t. before being quenched by addition of sodium citrate (10 mL, sat. aq. solution) and EtOAc (10 mL) was added. The organic layer was washed subsequently with sodium citrate (10 mL, sat. aq. solution) and water (3 × 10 mL). After drying and removal of the volatile compounds the crude product was recovered as a yellow oil. Precipitation from MeOH solution by addition of pentane gave pure **S6** as a yellow powder (105 mg, quant.). ^{1}H NMR (400 MHz, $CDCl_3$) δ 8.65 (s, 2H), 8.55 (s, 2H), 8.39 (d, *J* = 8.1 Hz, 2H), 7.83 (d, *J* = 8.1 Hz, 2H), 7.72 (d, *J* = 8.0 Hz, 2H), 7.27 (d, *J* = 8.1 Hz, 2H), 4.77 (s, 4H), 4.66 (s, 4H), 4.62 (s, 4H), ^{13}C NMR (126 MHz, $CDCl_3$) δ = 158.9, 155.8, 148.8, 148.1, 136.7, 136.6, 133.5, 132.1, 121.1, 120.4, 70.0, 69.9, 64.2. HRESI-MS: *m/z* = 459.2024 $[M+H]^+$ (calcd. for $C_{26}H_{27}N_4O_4$, 459.2032).

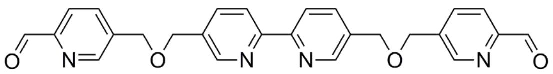

1

To a solution of oxalyl chloride (65 mg, 0.51 mmol) in DCM (1 mL) at -78 ºC was slowly added DMSO (88 mg, 1.12 mmol) in DCM (1 mL). The mixture was stirred for 15 min at -78 ºC before a solution of **S6** (110 mg, 0.23 mmol) in DCM (2 mL) was slowly added and the reaction was stirred for 1 h at -78 ºC. After addition of Et_3N (230 mg, 2.3 mmol) the mixture was stirred for a further 30 min at -78 ºC before being allowed to warm to 0 ºC and quenched with water (5 mL). The organic layer was washed with water (3 × 5 mL), then brine (5 mL), dried ($MgSO_4$), filtered under gravity and the solvent was removed in vacuo. The product was purified by flash

chromatography (SiO_2, 4:1, EtOAc: DCM) to give a colorless powder (76 mg, 72 %).^{1}H NMR (400 MHz, $CDCl_3$) δ 10.09 (s, 2H), 8.78 (s, 2H), 8.68 (s, 2H), 8.43 (d, *J* = 8.1 Hz, 2H), 7.99 (d, *J* = 8.0 Hz, 2H), 7.91 (d, *J* = 7.6 Hz, 2H), 7.86 (d, *J* = 8.1 Hz, 2H), 4.72 (s, 8H), ^{13}C NMR (101 MHz, $CDCl_3$) δ = 193.2, 155.8, 152.5, 149.3, 148.8, 138.4, 136.7, 136.2, 133.2, 121.7, 121.1, 70.5, 69.6, HRESI-MS: *m/z* = 455.1708 $[M+H]^+$ (calcd. for $C_{26}H_{23}N_4O_4$, 455.17079) and 477.1523 $[M+Na]^+$ (calcd. for $C_{26}H_{22}N_4O_4Na$, 477.1539).

SI-Section 3: Synthesis of Iron(II) Helicates

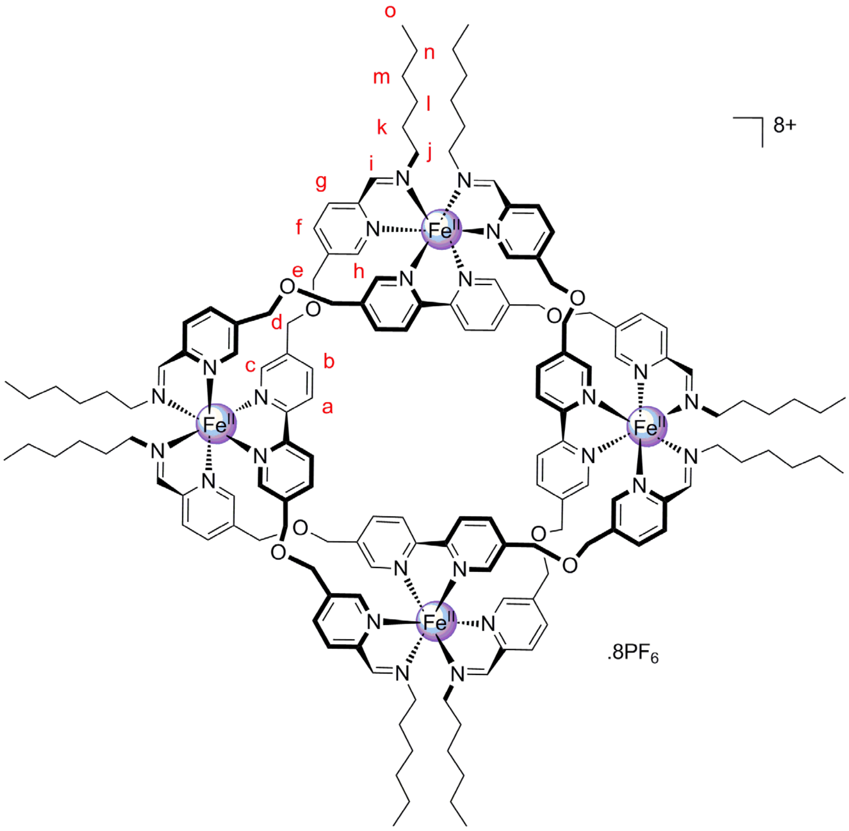

Synthesis of helicate 2

To a solution of dialdehyde **1** (10 mg, 22 µmol, 1 eq.) in DMSO-d$_6$ (5 mL) was added a solution of anhydrous $FeCl_2$ (250 µL of a 97 mM DMSO-d$_6$ solution, 24 µmol, 1.1 eq.) followed by a DMSO-d$_6$ solution of hexylamine (250 µL, 190 mM, 48 µmol, 2.2 eq.). The resulting dark purple reaction was thoroughly mixed and heated at 60 °C for one day. After cooling to room temperature, excess saturated aqueous KPF_6 was added (5 mL). A fine suspension of a purple material formed which was collected on Celite, thoroughly washed with water, ethanol, and diethylether. The purple solid was dissolved in acetonitrile and concentrated under reduced pressure to give **2** as a purple powder (10 mg, 47 %). ^{1}H NMR (500 MHz, CD_3CN) δ 9.22 (d, J = 8.3 Hz, 8H, H^a), 9.11 (s, 8H, H^i), 8.17 (d, J = 8.1 Hz, 8H, H^g), 7.86 (d, J = 8.2 Hz, 8H, H^f), 7.77 (s, 8H, H^c), 7.66 (d, J = 8.5 Hz, 8H, H^b), 7.35 (s, 8H, H^h), 4.76 (d, J = 14.2 Hz, 8H, H^e), 4.6 (m, 16H, H^d and H^e), 4.50 (d, J = 13.4 Hz, H^d), 3.44 (m, 8H, H^j), 3.02 (m, 8H, H^j), 1.57 (m, 8H, H^k), 1.47 (m, 8H, H^k), 1.18 (m, 16H, H^l), 1.09 (m, 32H, H^m and H^n), 0.86 (t, J = 7.3 Hz, 24H, H^o), ^{13}C NMR (126 MHz, CD_3CN) δ 172.6, 159.0, 157.2, 152.6, 152.1, 139.7, 139.4, 138.7, 136.4, 129.2, 124.2, 70.1, 69.5, 60.1, 31.8, 29.9,

27.0, 23.1, 14.2. HRESI-MS: *m/z* = 821.5311 $[M-4(PF_6)]^{4+}$ (calcd. for $C_{152}H_{192}F_{24}Fe_4N_{24}O_8P_4$, 821.5333).

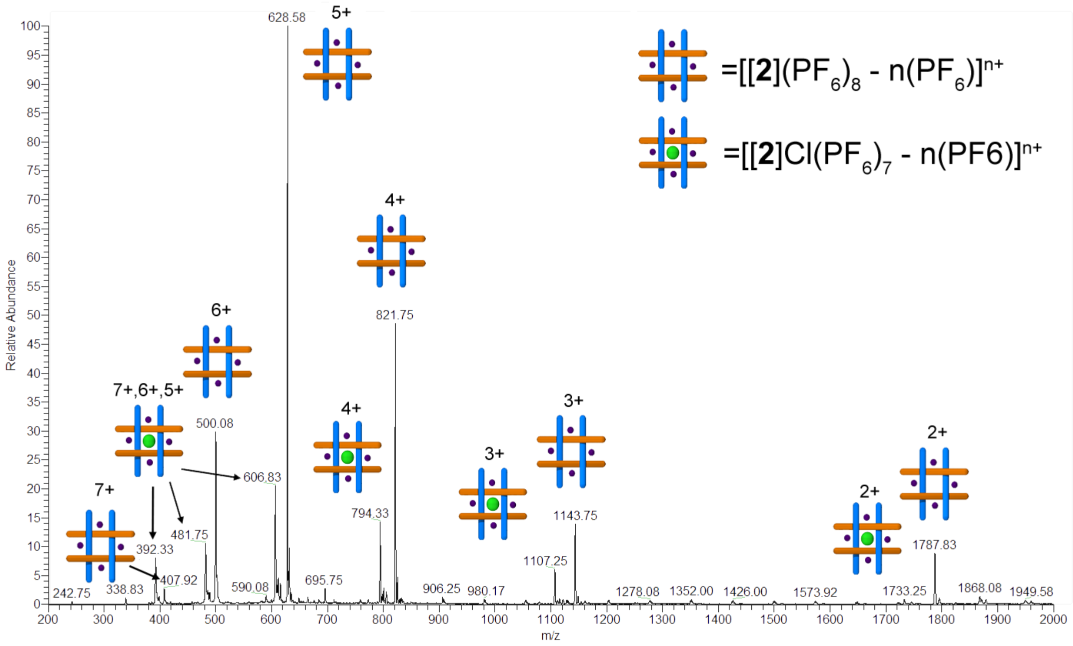

Figure S1. LRESI-MS analysis of **2**. Peaks corresponding to chloride containing and chloride-free helicates were observed.

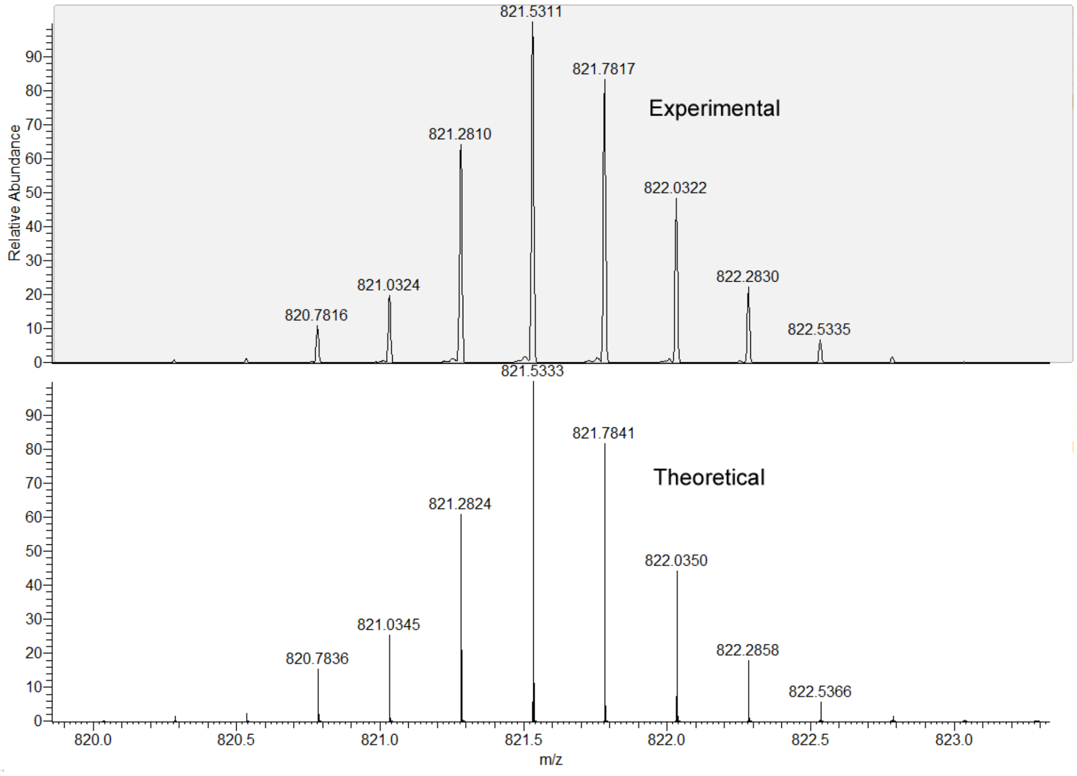

Figure S2. HRESI-MS analysis of $[\mathbf{2}(PF_6)_4]^{4+}$, indicating excellent correlation between the experimental (top) and theoretical (bottom) isotope patterns.

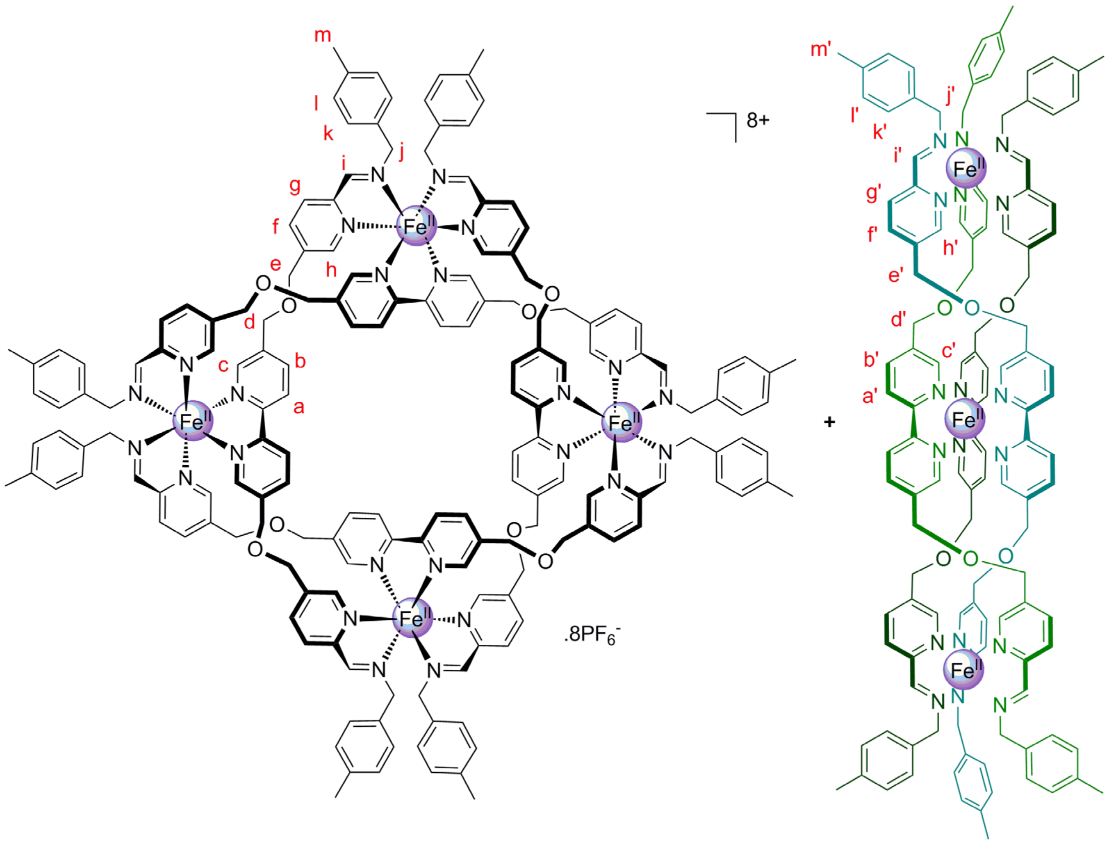

Synthesis of helicates 4 and 5

To a solution of dialdehyde **1** (10 mg, 22 μmol, 1 eq.) in DMSO-d_6 (12.5 mL) was added a solution of anhydrous $FeCl_2$ (250 μL of a 97 mM DMSO-d_6 solution, 24 μmol, 1.1 eq.) followed by a DMSO-d_6 solution of 4-methylbenzylamine (250 μL, 190 mM, 48 μmol, 2.2 eq.). The resulting dark purple reaction was thoroughly mixed and heated at 60 °C for one day. After cooling to room temperature, excess saturated aqueous KPF_6 was added (5 mL). A fine suspension of a purple material formed which was collected on Celite, thoroughly washed with water, ethanol, and diethylether. The purple solid was dissolved in acetonitrile and concentrated under reduced pressure to give a 1:1 mixture of **4** and **5** (9 mg, 46 % combined yield of helicates). The effects of concentration on this reaction were investigated see Figure S14.

Data for **4** (tetramer): ^{1}H NMR (500 MHz, CD_3CN) δ 9.20 (d, J = 8.4 Hz, 8H, H^a), 8.60 (s, 8H, H^i), 7.95 (d, J = 8.1 Hz, 8H, H^g), 7.84 (d, J = 8.4 Hz, 8H, H^f), 7.77 (s, 8H, H^c), 7.65 (d, J = 8.6 Hz, 8H, H^b), 7.36 (s, 8H, H^h), 7.18 (d, J = 7.8 Hz, 16H, H^l), 6.82 (d, J = 8.1 Hz, 16H, H^k), 4.85 (d, J = 14.3 Hz, 8H, H^e), 4.66 (m, 24H, H^d and H^e and H^j), 4.52 (d, J = 13.4 Hz, 8H, H^d), 4.21 (d, J = 15.4 Hz, 8H, H^j), 2.35 (s, 24H, H^m), ^{13}C NMR (126 MHz, CD_3CN) δ = 173.1, 159.1, 157.8, 152.5, 152.2, 140.0, 139.7,

138.6, 136.4, 130.9, 130.7, 130.3, 129.9, 128.0, 125.2, 70.2, 69.6, 64.3, 21.3, HRESI-MS: *m/z* = 659.9837 $[M-5(PF_6)]^{5+}$ (calcd. for $C_{168}H_{160}F_{18}Fe_4N_{24}O_8P_3$, 659.9829).

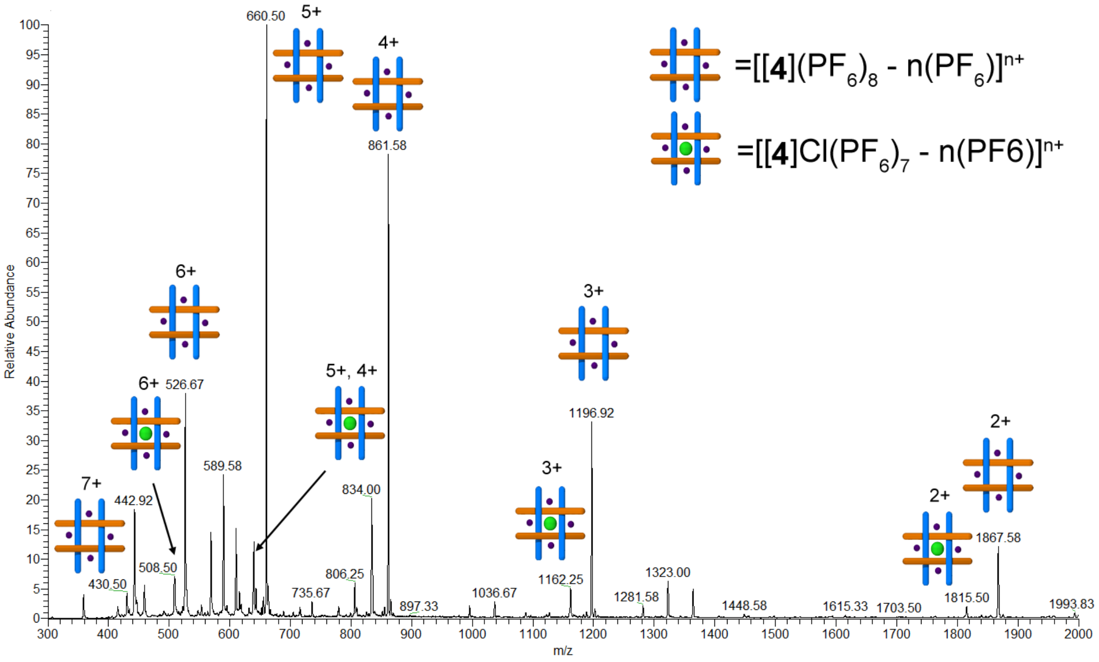

Figure S3. LRESI-MS analysis of **4**. Peaks corresponding to chloride containing and chloride free helicates were observed. A small amount of linear triple helicate **5** was also evident (see Figure S5).

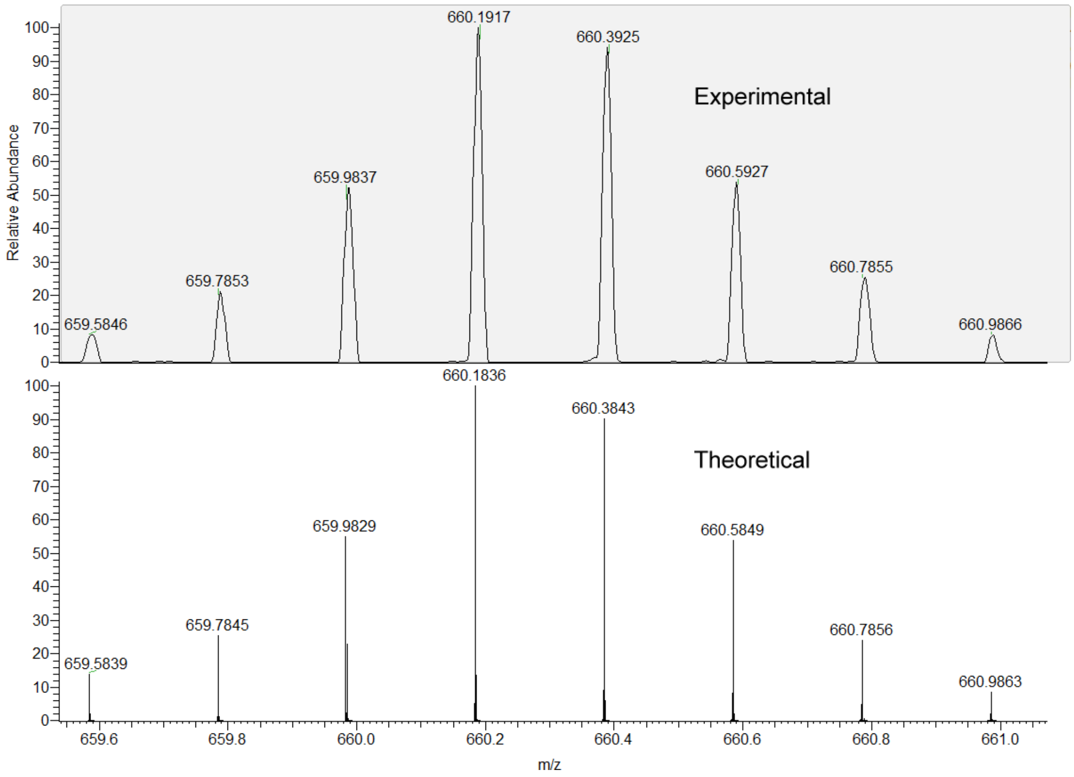

Figure S4. HRESI-MS analysis of $[\mathbf{4}(PF_6)_3]^{5+}$, indicating excellent correlation between the experimental (top) and theoretical (bottom) isotope patterns.

Data for **5** (linear triple helicate): ^{1}H NMR (500 MHz, CD_3CN) δ 8.34 (m, 12H, H^a and H^i), 7.95 (d, J = 8.1 Hz, 6H, H^b), 7.80 (d, *J* = 7.8 Hz, 6H, H^f), 7.46 (d, J = 8.0 Hz, 6H, H^g), 6.99 (s, 6H, H^c), 6.82 (d, *J* = 7.8 Hz, 12H, H^l), 6.62 (d, *J* = 7.9 Hz, 12H, H^k), 6.51 (d, *J* = 17.8 Hz, 6H, H^h), 5.30 (d, *J* = 13.7 Hz, 6H, H^j), 4.85 (d, *J* = 14.3 Hz, 6H, H^j), 4.40 (m, 12H, H^e), 4.30 (d, *J* = 19.7 Hz, 12H, H^d), 2.26 (s, 18H, H^m), ^{13}C NMR (126 MHz, CD_3CN) δ = 174.3, 153.4, 139.5, 139.2, 137.3, 131.7, 130.5, 130.1, 129.4, 129.1, 127.4, 126.0, 124.5, 122.2, 121.4, 70.2, 69.6, 66.2, 21.1. HRESI-MS: *m/z* = 458.9461 $[M-5(PF_6)]^{5+}$ (calcd. for $C_{126}H_{120}F_6Fe_3N_{18}O_6P$, 458.9467).

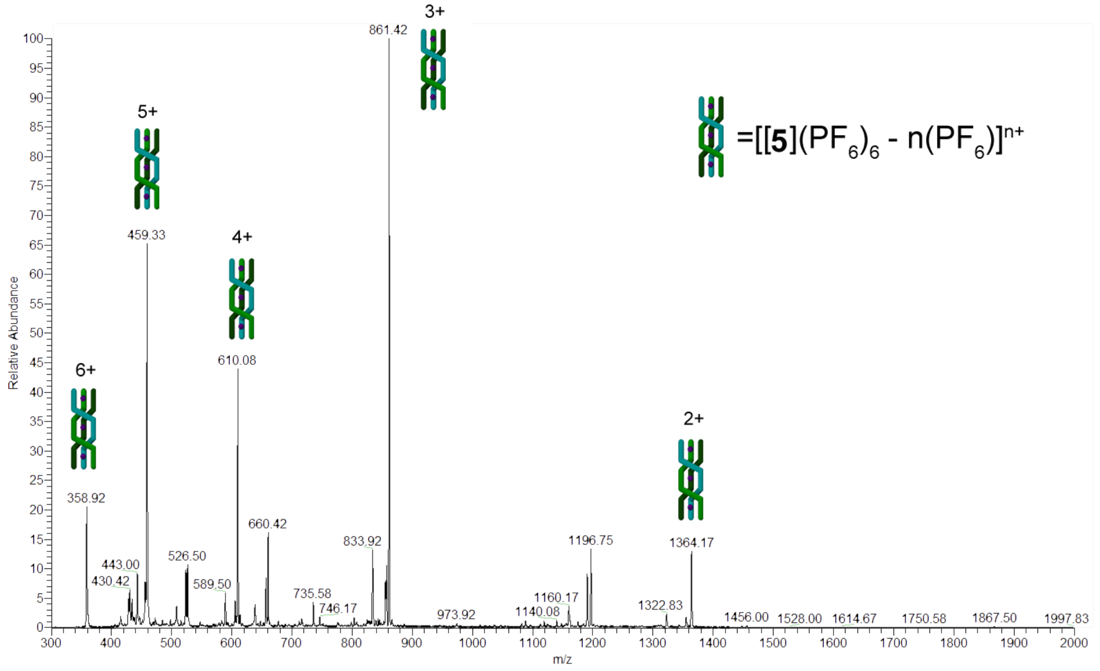

Figure S5. LRESI-MS analysis of **5**. A small amount of circular helicate **4** was also evident (see Figure S3).

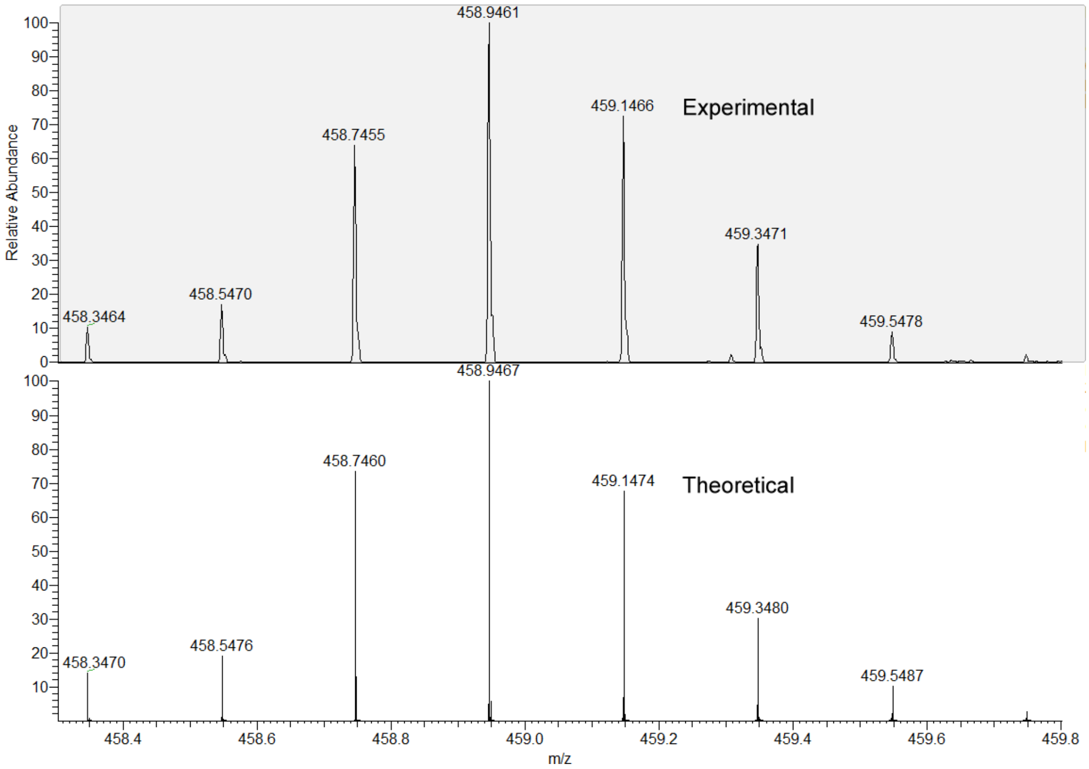

Figure S6. HRESI-MS analysis of [**5**(PF_6)]$^{5+}$, indicating excellent correlation between the experimental (top) and theoretical (bottom) isotope patterns.

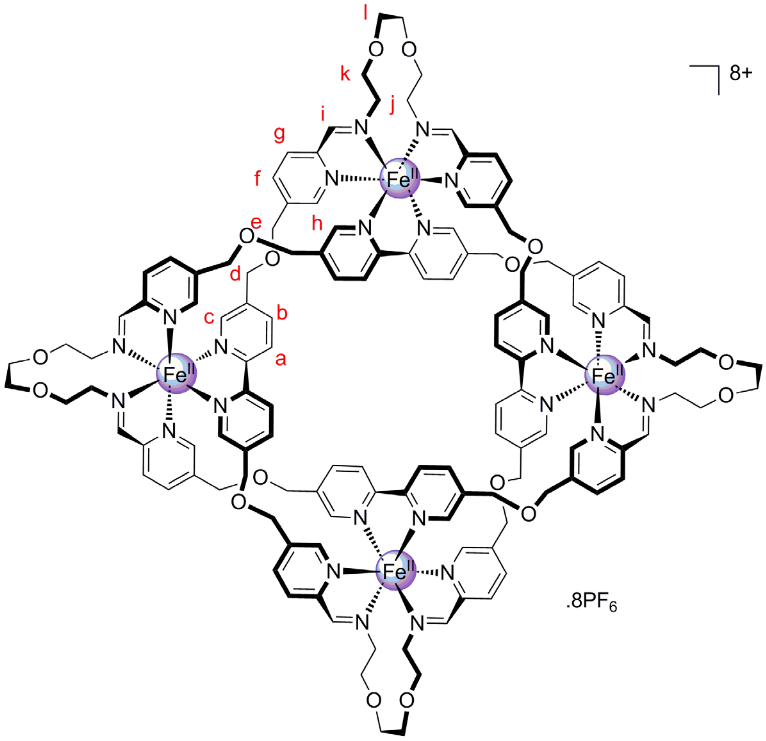

Synthesis of Solomon link 6

To a solution of dialdehyde **1** (10 mg, 22 µmol, 1 eq.) in DMSO-d_6 (5 mL) was added a solution of anhydrous $FeCl_2$ (250 µL of a 97 mM DMSO-d_6 solution, 24 µmol, 1.1 eq.) followed by a DMSO-d_6 solution of 2,2'-(ethylenedioxy)bis(ethylamine) (250 µL, 97 mM, 24 µmol, 1.1 eq.). The resulting dark purple reaction was thoroughly mixed and heated at 60 °C for 24 h. After cooling to room temperature, excess saturated aqueous KPF_6 was added (5 mL). A fine suspension of a purple material formed which was collected on Celite, thoroughly washed with water, ethanol (soluble in 1:1 ethanol:water), and diethylether. The purple solid was dissolved in acetonitrile and concentrated under reduced pressure to give **5** as a purple powder (15 mg, 75 %). ^{1}H NMR (500 MHz, CD_3CN) δ 9.17 (d, J = 8.4 Hz, 8H, H^a), 9.07 (d, J = 14.8 Hz, 8H, H^i), 8.20 (d, J = 8.0 Hz, 8H, H^g), 7.87 (d, J = 8.0 Hz, 8H, H^f), 7.80 (s, 8H, H^c), 7.62 (d, J = 8.0 Hz, 8H, H^b), 7.36 (s, 8H, H^h), 4.77 (d, J = 13.8 Hz, 8H, H^e), 4.66 (m, 16H, H^e and H^d), 4.52 (d, J = 13.5 Hz, 8H, H^d), 4.10 (t, J = 10.1 Hz, 8H, H^j), 3.64 (m, 16H, $H^k$and H^l), 3.35 (d, J = 9.8 Hz, 8H, H^l), 3.23 (d, J = 13.3 Hz, 8H, H^j), 2.92 (m, 8H, H^k), ^{13}C NMR (126 MHz, CD_3CN) δ 175.6, 158.8, 157.9, 152.6, 150.5, 140.0, 139.9, 138.5, 136.6, 129.6, 125.1, 71.4, 70.2, 69.5, 69.1, 61.0, HRESI-MS: *m/z* = 584.9368 $[M-5(PF_6)]^{5+}$ (calcd. for $C_{128}H_{136}F_{18}Fe_4N_{24}O_{16}P_3$, 584.9379).

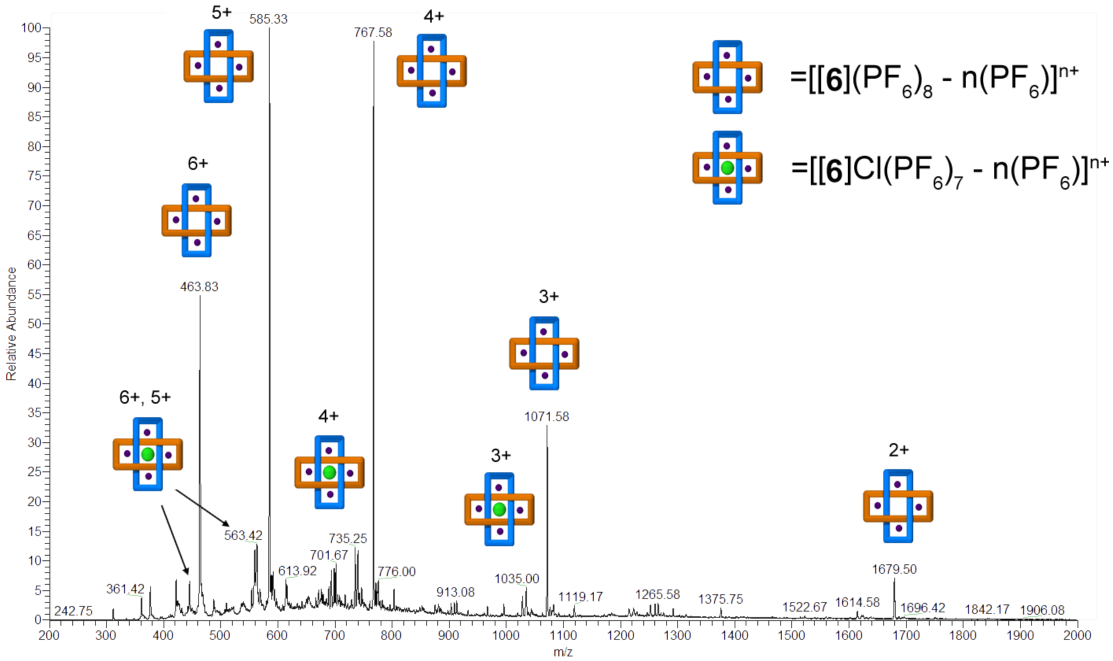

Figure S7. LRESI-MS analysis of **6**. Peaks corresponding to chloride-containing and chloride-free helicates were observed.

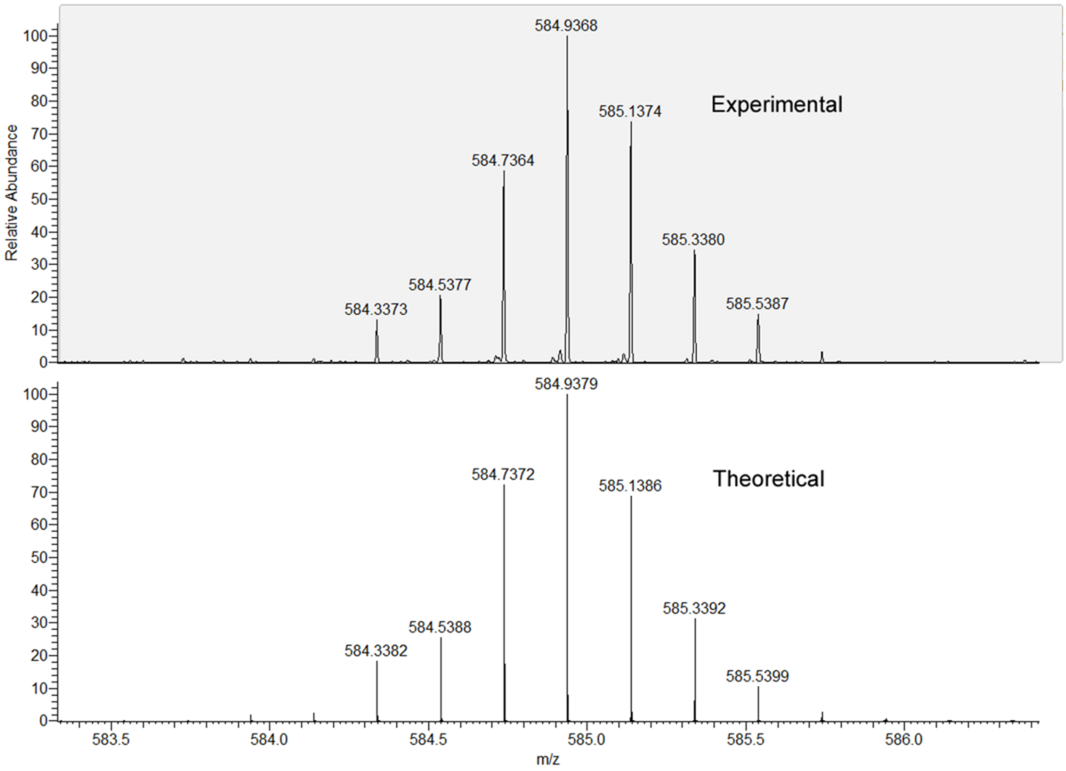

Figure S8. HRESI-MS analysis of $[\mathbf{6}(PF_6)_3]^{5+}$, indicating excellent correlation between the experimental (top) and theoretical (bottom) isotope patterns.

SI-Section 4: Effect of $FeCl_2$ stoichiometry on the formation of circular helicate 2

A solution of dialdehyde **1** (5 mg, 11 μmol, 5 eq.) in DMSO-d_6 (2.5 mL) was separated into five separate samples of 0.5 mL. To each sample was added varying amounts of a 97 mM DMSO-d_6 solution of anhydrous $FeCl_2$, as shown in the table below, followed by a DMSO-d_6 solution of hexylamine (25 μL, 190 mM, 4.8 μmol, 2.2 eq.).

Sample	Volume of $FeCl_2$ solution (μL)	Amount of $FeCl_2$ (μmol)	Equivalents (wrt dialdehyde 1)
1	20	2.0	0.9
2	25	2.4	1.1
3	50	4.8	2.2
4	75	7.3	3.3
5	100	9.7	4.4

The resulting dark purple reactions were thoroughly mixed and heated at 60 °C for one day. After cooling to room temperature, the following work up was performed on each sample separately: excess saturated aqueous KPF_6 was added (5 mL). A fine suspension of a purple material formed which was collected on Celite, thoroughly washed with water, a little ethanol (soluble), and diethylether. The purple solids were dissolved in acetonitrile and concentrated under reduced pressure to give **2** as a purple powder. Each sample was dissolved in CD_3CN (0.5 mL) and relative yields analyzed by ^{1}H NMR (Figure S9). A preparatory synthesis was carried out following the above procedure using 10 mg of **1** (22 μmol), and 4.0 eq of $FeCl_2$, gave 15 mg of **2** (71 %).

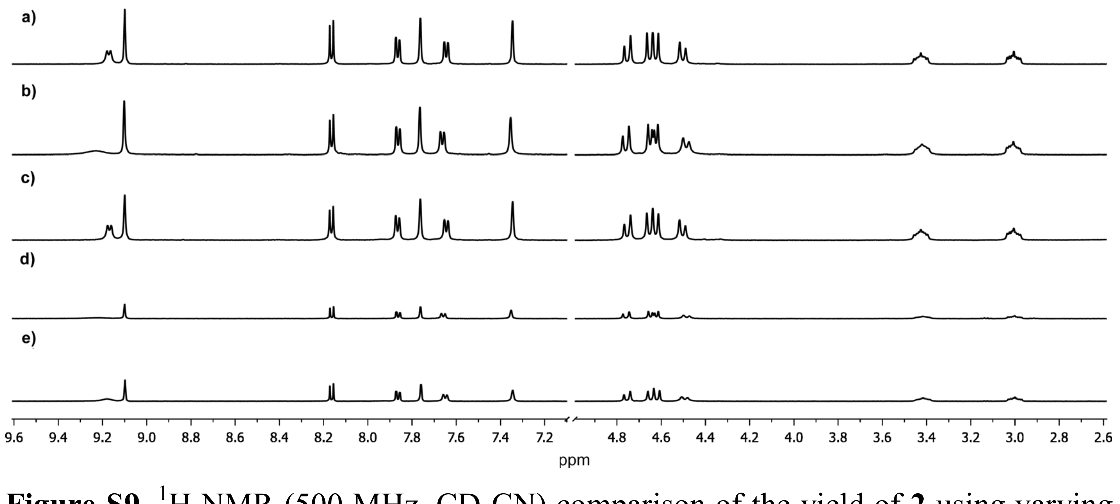

Figure S9. ^{1}H NMR (500 MHz, CD_3CN) comparison of the yield of **2** using varying amounts of $FeCl_2$ (a) 4.4 eq. (b) 3.3 eq. (c) 2.2 eq. (d) 1.1 eq. (e) 0.9 eq.

SI- Section 5: Effect of $AgPF_6$ addition to remove residual chloride

A small contamination of chloride anions still present after anion exchange causes the signal of H^a to become very broad. Addition of small amounts of $AgPF_6$ caused a dramatic sharpening of the peak as seen in Figures S10-S12. All other signals were unaffected by the addition. A small quantity of $AgPF_6$ (~0.1 eq) was added in CD_3CN solution to samples of **2**, **4/5** and **6**. ^{1}HNMR spectra were recorded before and after addition.

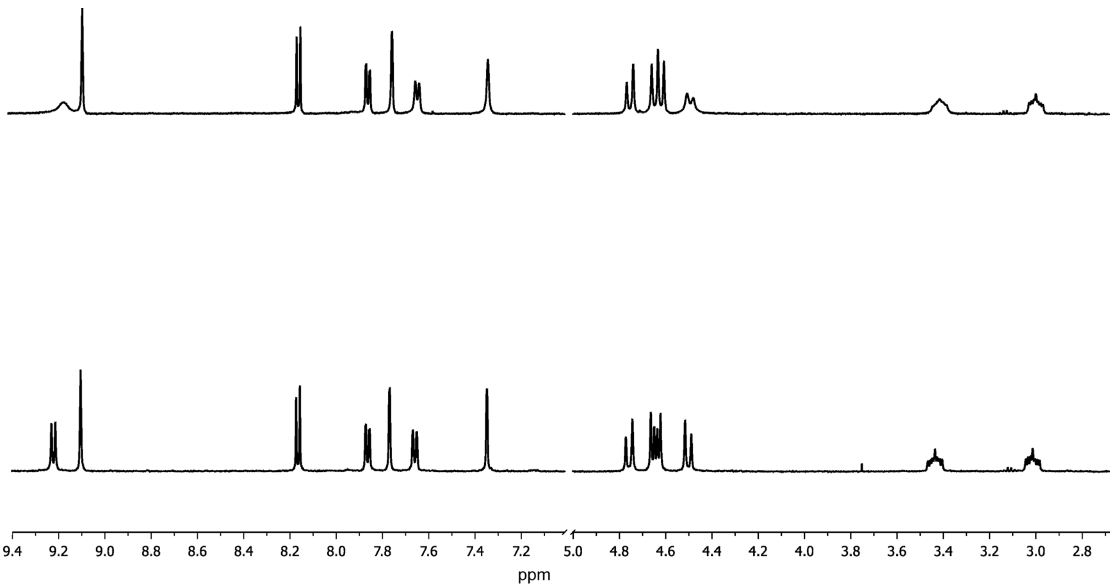

Figure S10. ^{1}H NMR (500 MHz, CD_3CN) of **2** in the absence (top) and presence (bottom) of ~0.1 equiv. $AgPF_6$.

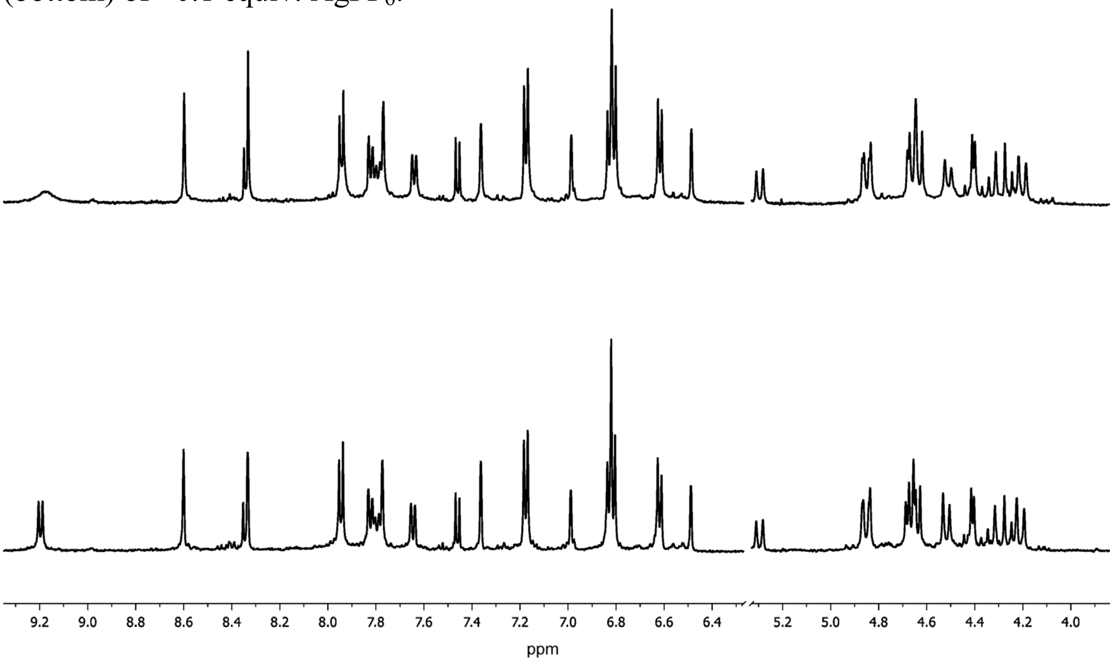

Figure S11. ^{1}H NMR (500 MHz, CD_3CN) of **4/5** in the absence (top) and presence (bottom) of ~0.1 equiv. $AgPF_6$.

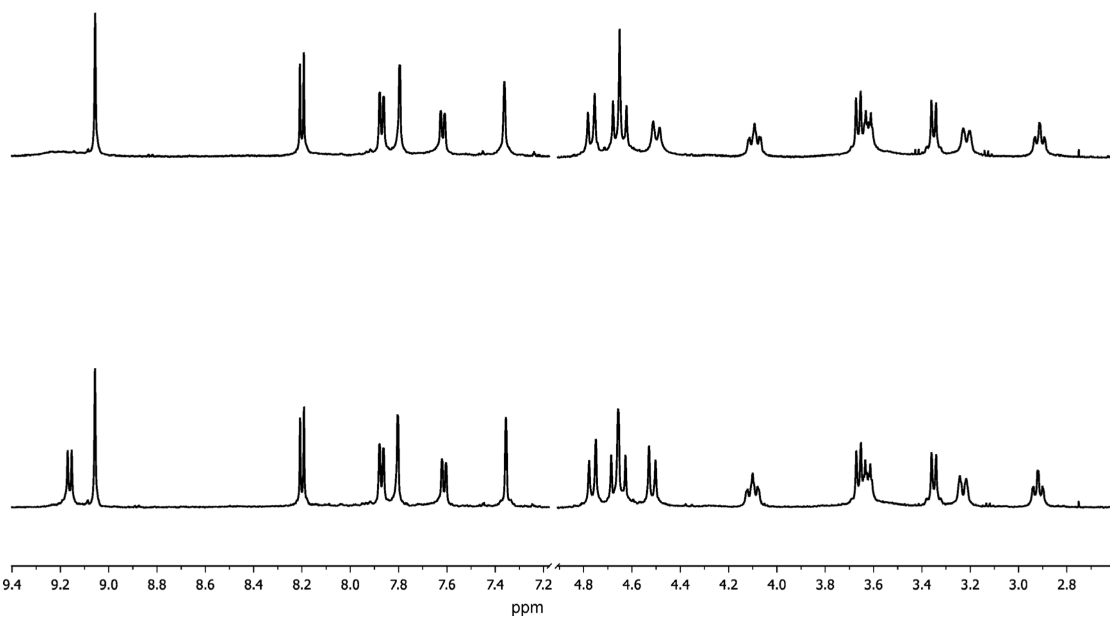

Figure S12. ^{1}H NMR (500 MHz, CD_3CN) of **6** in the absence (top) and presence (bottom) of ~0.1 equiv. $AgPF_6$.

SI-Section 6: The use of different iron(II) sources

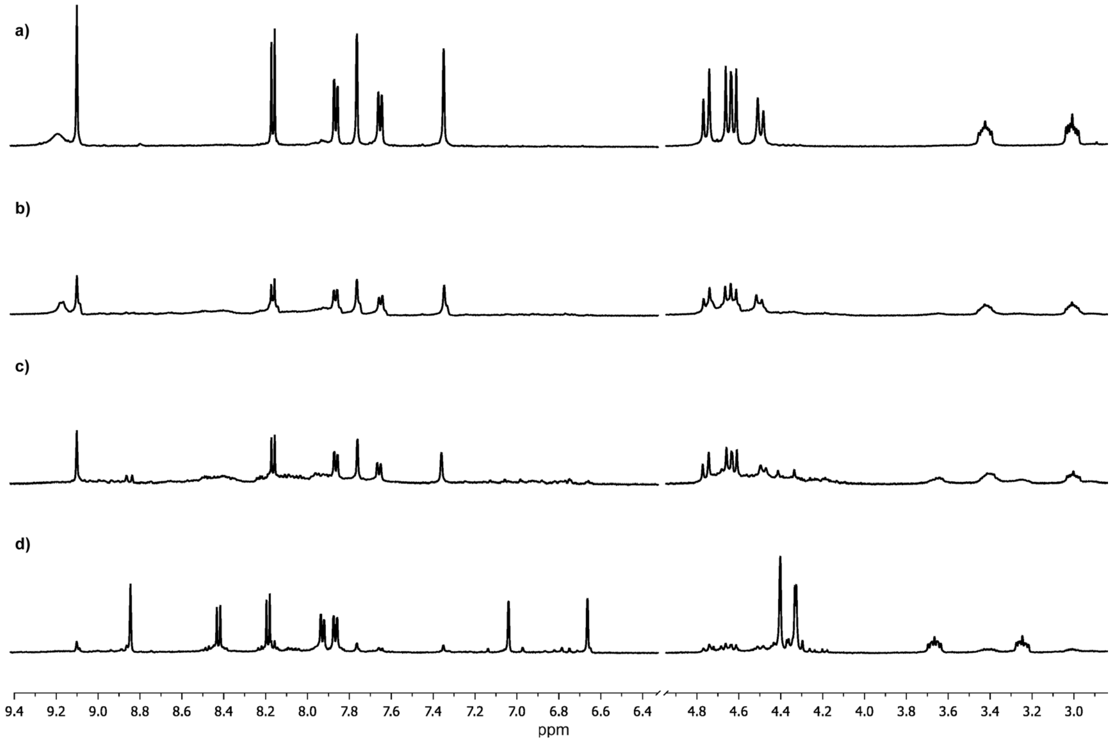

Figure S13. ^{1}H NMR (500 MHz, CD_3CN) analysis after work up of **2** prepared from (a) $FeCl_2$, (b) $Fe(BF_4)_2$, (c) $Fe(ClO_4)_2$, (d) $FeBr_2$ (in this case linear triple helicate **3** was formed with minor peaks corresponding to **2**, see main paper).

A stock solution of dialdehyde **1** (4 mg, 8.8 µmol, 4 eq. in 2 mL DMSO) was separated into four 0.5 mL samples. To each sample 25 µL of a DMSO solution (97 mM, 2.4 µmol, 1.1 eq.) of an iron(II) salt ($FeCl_2$, $Fe(BF_4)_2$, $Fe(ClO_4)_2$, $FeBr_2$ (added in DMF)) was added. A DMSO-d_6 solution of 1-hexylamine (25 µL, 190 mM, 4.8 µmol, 2.2 eq.) was added to each separate sample. All samples were heated at 60 ºC overnight. After cooling to room temperature, the following work up was performed on each sample separately: excess saturated aqueous KPF_6 was added (5 mL). A fine suspension of a purple material formed which was collected on Celite, thoroughly washed with water, ethanol, and diethylether. The purple solid was dissolved in acetonitrile and concentrated under reduced pressure. Each sample was analyzed by ^{1}H NMR see Figure S13.

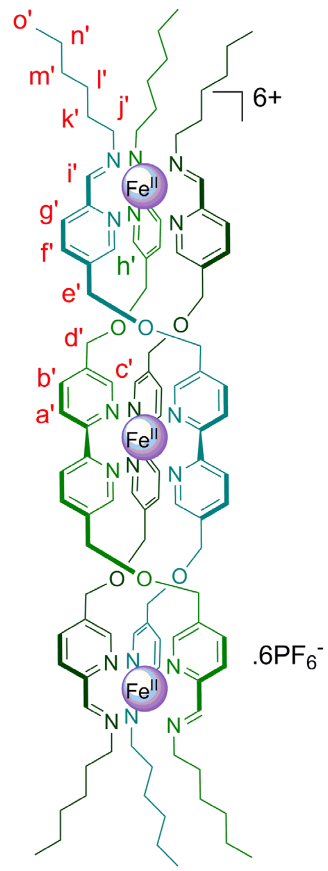

Data for triple helicate **3**: ^{1}H NMR (500 MHz, CD_3CN) δ 8.85 (s, 6H, $H^{i'}$), 8.42 (d, *J* = 8.3 Hz, 6H, $H^{a'}$), 8.18 (d, *J* = 8.0 Hz, 6H, $H^{g'}$), 7.93 (d, *J* = 6.9 Hz, 6H, $H^{f'}$), 7.87 (d, *J* = 8.1 Hz, 6H, $H^{b'}$), 7.04 (s, 6H, $H^{c'}$), 6.66 (s, 6H, $H^{h'}$), 4.40 (m, 12H, $H^{e'}$), 4.33 (m, 12H, $H^{d'}$), 3.67 (m, 6H, $H^{j'}$), 3.25 (m, 6H, $H^{j'}$), 1.59 (m, 12H, $H^{k'}$), 1.21 (m, 36H, $H^{l'}$, $H^{m'}$ and $H^{n'}$), 0.85 (t, *J* = 7.1 Hz, 18H, $H^{o'}$), ^{13}C NMR (126 MHz, CD_3CN) δ = 171.5, 159.1, 158.7, 140.3, 139.5, 139.3, 137.1, 137.0, 129.3, 124.6, 122.3, 70.4, 70.3, 60.4, 32.1, 30.1, 26.8, 23.1, 14.2.

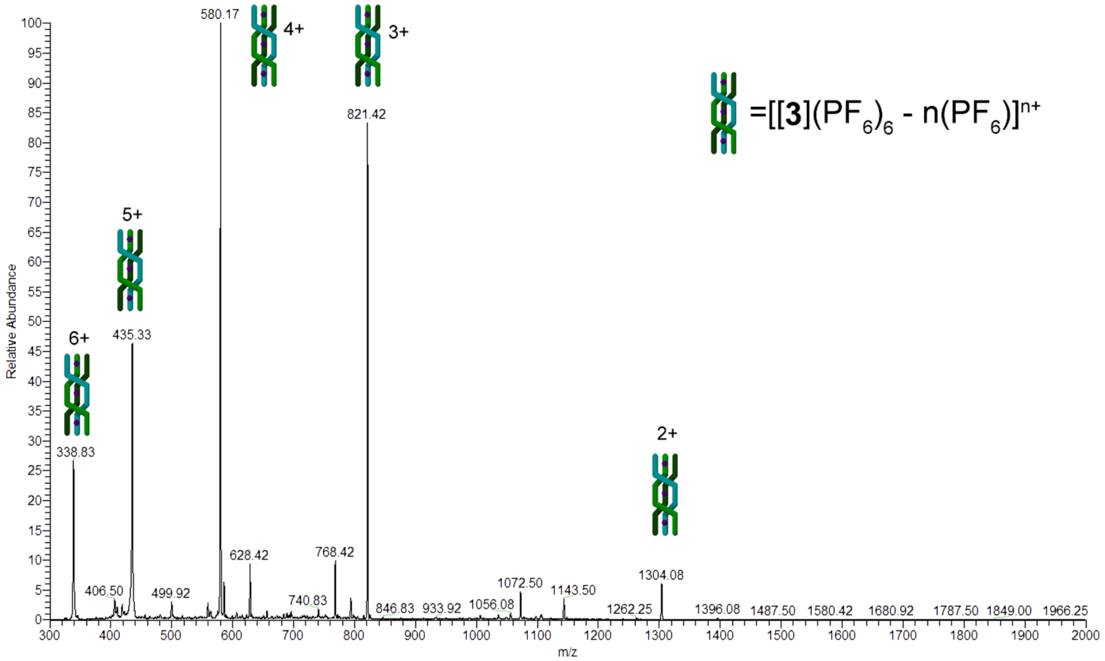

Figure S14: LRESI-MS analysis of **3**. A small amount of circular helicate **2** was evident (see Figure S1).

SI-Section 7: Effect of concentration on the formation of helicates using 4-methylbenzyl amine

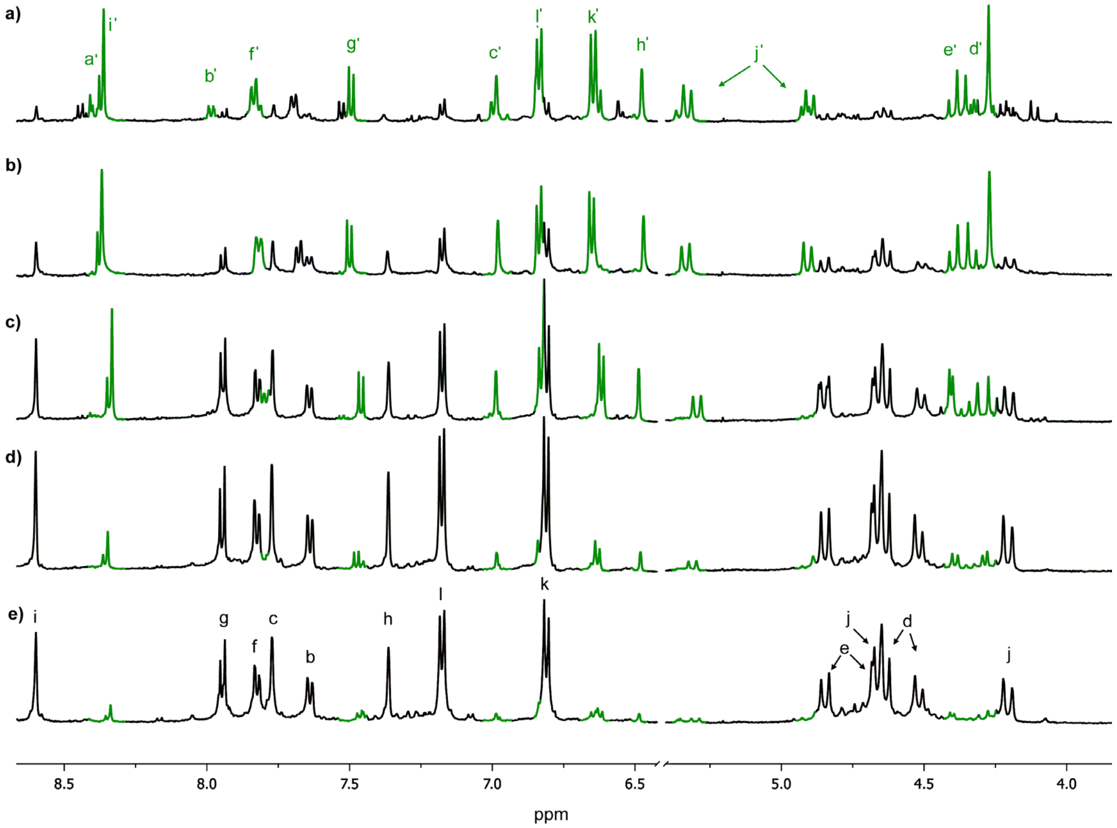

Figure S15. ^{1}H NMR(500 MHz, CD_3CN). Product distribution of M_3L_3 (green) vs M_4L_4 (black) helicates at different concentrations (a) 0.55 mM, (b) 1.1 mM, (c) 2.2 mM, (d) 4.4 mM, (e) 8.8 mM. Carrying reactions at higher or lower concentrations lead to the formation of additional uncharacterized products.

To a solution of dialdehyde **1** (5 mg, 11.0 µmol, 5 eq.) in DMSO-d_6 (1.25 mL) was added a solution of anhydrous $FeCl_2$ (125 µL of 97 mM, 12.1 µmol, 5.5 eq.). This mixture was stirred to ensure complete dissolution of the aldehyde then separated into five samples of 0.25 mL each. Each sample was diluted to a different concentration by addition of DMSO-d_6: no addition (8.8 mM), 0.25 mL (4.4 mM), 0.75 mM (2.2 mM), 1.75 mL (1.1 mM), 3.75 mL (0.55 mM). To each sample was added a DMSO-d_6 solution of 4-methylbenzylamine (25 µL of a 190 mM, 4.8 µmol, 2.2 eq.). The resulting dark purple solutions were thoroughly mixed and heated at 60 °C for one day. After cooling to room temperature, the following work up was performed on each sample separately: excess saturated aqueous KPF_6 was added (5 mL). A fine suspension of a purple material formed which was collected on Celite, thoroughly washed with water, ethanol, and diethylether. The purple solid was dissolved in acetonitrile and concentrated under reduced pressure. The products were analyzed by

^{1}H NMR, shown in Figure S15, addition characterization was in line with that reported above. When the reaction was carried out at concentrations outside the range reported above addition polymeric and oligomeric material was found to contaminate the recovered products.

SI-Section 8: X-Ray Crystallography

Crystal data for Solomon link **6**

[$C_{128}H_{136}F_{48}Fe_4N_{24}O_{16}P_8$ 8(PF_6).4(CH_3NO_2).3½(CH_4O).½($C_4H_{10}O$).2½H_2O]: M_r = 4088.19, triclinic, space group P-1, T = 100 K, a = 20.596(10), b = 22.540(9), c = 25.460(8) Å, α = 65.423(19), β = 76.01(2), γ = 88.03(2)°, V = 10400(8) Å^3, Z = 2, ρ = 1.248 g cm^{-3}, total data 43797, independent reflections 14073 (R_{int}=0.144), μ = 0.441 mm^{-1}, 2339 parameters, R_1 = 0.1538 for I>2σ(I) and wR2 = 0.3562. The crystal structure has been deposited at the Cambridge Crystallographic Data Centre, deposition number CCDC-931375.

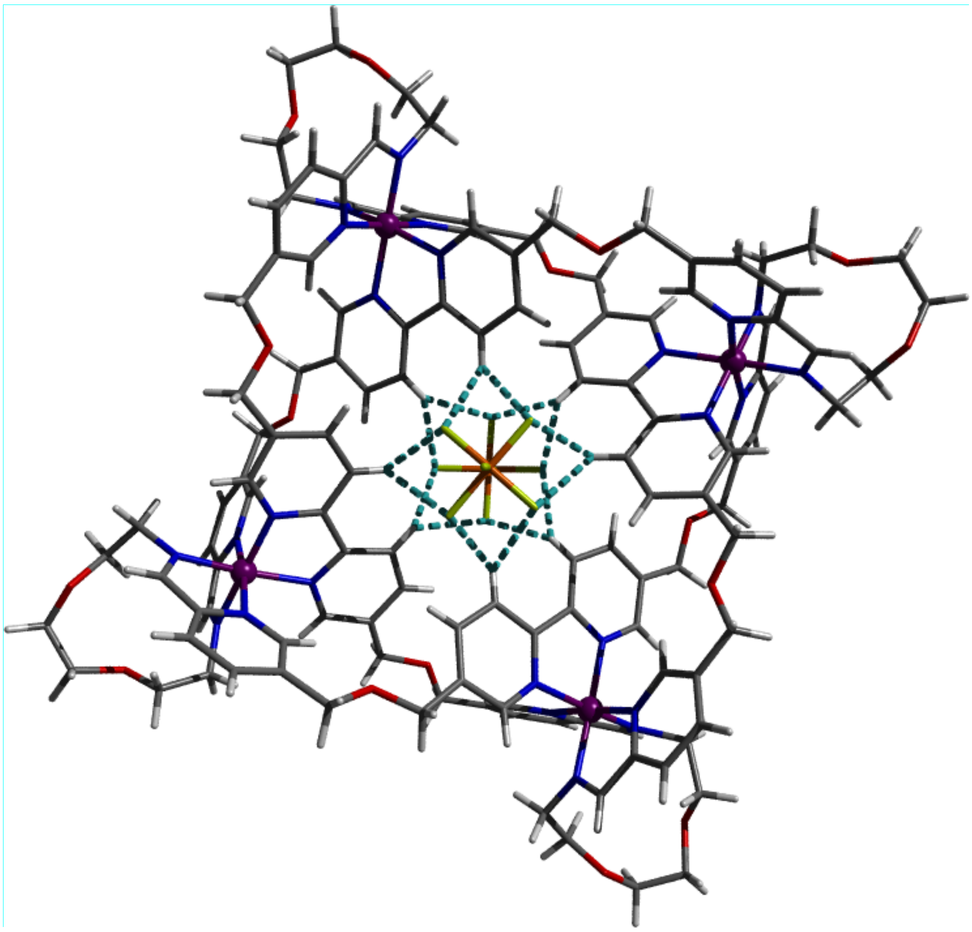

Figure S16. X-Ray crystal structure of Solomon link **6** highlighting the interactions between H^a and the PF_6 counter-ions (shown in green). CH-F contact distances (Å):

2.72, 2.82, 2.74, 2.65, 2.78, 2.89, 2.99, 2.74, 2.96, 2.70, 2.86, 2.88, 2.85, 3.15, 2.95, 2.87.

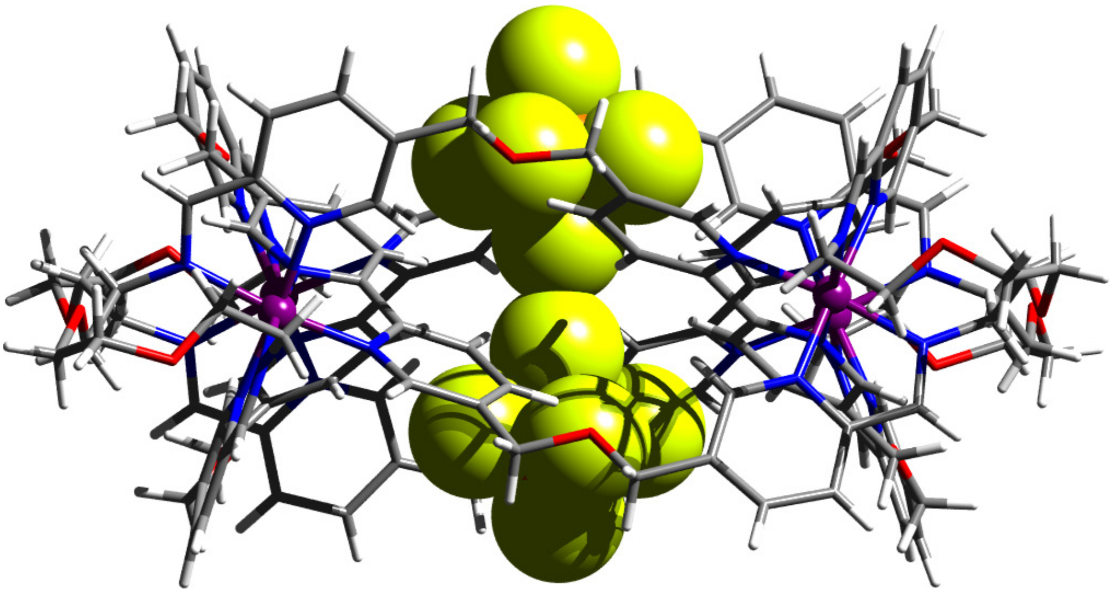

Figure S17. Side view of **6** with PF_6 (shown in space-filling model) counter ions, above and below the plane of the four iron(II) ions.

References

S1. A. Landa, A. Minkkil, G. Blay, K. A. Jørgensen, *Chem. Eur. J.* **2006**, *12*, 3472–3483.

S2. R. N. Guthikonda, L. D. Cama, M. Quesada, M. F. Woods, T. N. Salzmann, B. G. Christensen, *J. Med. Chem.* **1987**, *30*, 871–880.

S3. N. D. Jabre, T. Respondek, S, A. Ulku, N, Korostelova, J J. Kodanko, *J. Org. Chem.* **2010**, *75*, 650–659.

S4. Y. An, M. L. Tong, L. N. Ji, Z. W Mao, *Dalton Trans.* **2006**, 2066–2071.
